# Processing and Formulation Optimization of Mandarin Essential Oil-Loaded Emulsions Developed by Microfluidization

**DOI:** 10.3390/ma13163486

**Published:** 2020-08-07

**Authors:** Jenifer Santos, Nuria Calero, Luis Alfonso Trujillo-Cayado, María José Martín-Piñero, José Muñoz

**Affiliations:** 1 Departamento de Ingeniería Química, Escuela Politécnica Superior, Universidad de Sevilla c/Virgen de África 7, E41011 Sevilla, Spain; ltrujillo@us.es; 2 Departamento de Ingeniería Química, Facultad de Química, Universidad de Sevilla c/P. García González 1, E41012 Sevilla, Spain; mjmartin@us.es (M.J.M.-P.); jmunoz@us.es (J.M.)

**Keywords:** emulsion, guar gum, mandarin essential oil, microfluidization, rheology

## Abstract

Emulsions can be used as delivery systems for bioactive ingredients for their incorporation in food products. Essential oils are natural compounds found in plants that present antioxidant and antimicrobial activity. Therefore, the main goal of this work was to develop emulsions, containing mandarin essential oil stabilized by two food-grade surfactants and guar gum, and to evaluate their physical stability. The initial droplet size of emulsions developed by microfluidization was optimized, obtaining diameters below one micron regardless of the processing conditions. However, the emulsion processed at 25,000 psi and one pass exhibited the lowest mean droplet sizes and polidispersity, and therefore, a higher stability. Different ratios of Tween 80 and Span 80 were assessed as stabilizers. Results obtained indicated that the ratio of surfactants had a significant effect on the mean droplet sizes, physical stability, and rheological properties. Thus, we found that the optimum ratio of surfactants was 75/25 (Tween80/Span80) on account of the lowest droplet mean diameters, lack of coalescence, and a low creaming rate. The rheological characterization of the stable emulsions showed a shear thinning flow behavior, and G″ (loss modulus) values higher than G′ (storage modulus) values, in all the frequency range. The rheological behavior may be governed by the guar gum, which was confirmed by field emission scanning electron microscopy (FESEM). This research can be considered as the starting point for future applications of mandarin essential oil in emulsions, which can be incorporated in products as food preservatives.

## 1. Introduction

In recent years, the importance of using essential oils in the formulation of antimicrobial products and their role as natural biocides has increased [1,2]. Essential oils are mixtures of volatile aromatic products that can be extracted from different plants and/or herbs. Most essential oils are generally recognized as safe (GRAS) substances [3]. Due to their antibacterial [4], antifungal [5], antiviral [6], and insecticidal activities [7], essential oil-based products are used in the food, agrochemical, pharmaceutical, cosmetic, and medical fields. The hydrophobicity of essential oils is one of the most important factors determining their antimicrobial activity [8]. Furthermore, the lipophilic character of essential oils means that these solvents cannot be directly dispersed into an aqueous phase. This fact, in combination with their antioxidant activity [9], means that they are suitable for being encapsulated into a variety of different colloidal delivery systems, with the most common being emulsions [10]. These colloidal systems consist of two immiscible liquids, where one liquid is dispersed into the other in the form of small droplets. Therefore, several works have focused on the encapsulation of essential oils in emulsions and emulgels. Recent studies include the production of these delivery systems from numerous essential oils, such as cinnamon oil [11,12], thyme oil [13,14], lemongrass oil [15], rosemary oil [16], fennel oil [17,18], oregano oil [19], or mixtures of clove and melaleuca essential oils [20]. The most important application of these emulsions and emulgels is as food preservatives, based on the antimicrobial activity of essential oils. In addition, essential oils seem to maintain their biological properties after emulsification processing [21].

Mandarin essential oil could be considered a suitable alternative to chemical additives for use in the food industry. Nanoemulsions formulated with mandarin essential oil have been used as an option to prevent toxic compounds in seafood [22], and to reduce the growth of *Aspergillus flavus* [23]. Emulsions are not thermodynamically stable systems that can undergo a variety of different destabilization mechanisms including sedimentation, creaming, coalescence, flocculation, and Ostwald ripening. However, these dispersed systems can show long physical stability with the correct selection of both processing and formulation. In emulsions, the stabilization of the oil droplets in the aqueous phase requires the use of surfactants. One important property of surfactants that should be taken into account in formulating emulsions is hydrophilic-lipophilic balance (HLB) value. Tween 80 and Span 80 are two surfactants, considered as food grade, with very different HLBs. The mixture of both surfactants can fine-tune the HLB value required for a specific oil. For this purpose, these surfactants were used as emulsifiers in the present work.

Furthermore, the physical stability of emulsions largely depends on the emulsification method used. There are several methods of forming emulsions, high-energy methods, such as microfluidization, being the most commonly used. However, the first approaches to forming mandarin essential oil-in-water emulsions were carried out by means of low energy methods [24]. Concerning high-energy methods, high-pressure valve homogenizers proved to be a good choice to prepare mandarin essential oil emulsions [25]. To the best of our knowledge, there have been no studies about the development of emulsions formulated with mandarin essential oil using a microfluidizer. However, there are different papers that prove the suitability of using a microfluidizer to prepare emulsions containing essential oils [26]. Furthermore, natural biopolymers are gaining importance for their use as thickening agents, in order to enhance the physical stability of emulsions and nanoemulsions. Guar gum is a biopolymer that has been used in a multitude of food systems [27].

The main goal of this study is to develop an optimal emulsion formulation containing mandarin essential oil as dispersed phase, and to simultaneously provide a useful guideline for food emulsion manufacturers. For that purpose, ensuring the extent of the impact microfluidization conditions have on an emulsion’s mean droplet diameters, and physical stability, will ensure the yield of a stable product. The optimum homogenization pressure and number of cycles were initially identified. Furthermore, taking into account the results obtained, the influence of different surfactant ratios was investigated. Guar gum was incorporated into the formulation in order to enhance the physical stability and rheological properties.

## 2. Materials and Methods

### 2.1. Materials

Bidah Chaumel (Murcia, Spain) supplied the ecological mandarin oil. Tween 80, Span 80, and guar gum powder were provided by Sigma Aldrich (San Luis, MO, USA). All systems studied were developed using distilled water.

### 2.2. Emulsions Development

First, the surfactant Tween 80 was added to distilled water and the surfactant Span 80 to the mandarin oil. In order to prepare the coarse emulsion (250 g), a Silverson L5M rotor-stator homogenizer (Silverson, Chesham, UK) was used at 4000 rpm for 30 s (while the addition of oil was taking place), and then at 6000 rpm for 30 s. The droplet size of the coarse emulsion was reduced using a microfluidization device (Microfluidizer M110P, Microfluidics, Westwood, MA, USA) at the homogenization pressure selected. In the first part of this study, different homogenization pressures were studied. A scheme of microfluidization was reported by Jafari, 2019 [26].

Once the nanoemulsion was prepared, guar gum solution was added. Previously, a guar gum solution stock (1 wt.%) was prepared using an IKA-Visc MR-D1 (IKA-Werke, Staufen, Germany) in combination with a saw-tooth type rotor at 600 rpm for 4 h. Each final emulsion sample contained a mandarin essential oil concentration of 5 wt.%, a total surfactant concentration of 0.5 wt.%, a guar concentration of 0.25 wt.%, and distilled water.

### 2.3. Droplet Size Distributions

In order to obtain droplet size distributions and droplet mean diameters, laser diffraction measurements were conducted using a Malvern Mastersizer 2000 (Malvern Instruments, Worcestershire, UK). Sauter diameter (*D*_3,2_), volumetric diameter (*D*_4,3_), and span parameter were calculated as follows:

(1) $$D_{3,2}=\overset{N}{\underset{i=1}{\sum }}n_{i}d_{i}^{3}/\overset{N}{\underset{i=1}{\sum }}n_{i}d_{i}^{2}\;$$

(2) $$D_{4,3}=\overset{N}{\underset{i=1}{\sum }}n_{i}d_{i}^{4}/\overset{N}{\underset{i=1}{\sum }}n_{i}d_{i}^{3}\;$$

where *d_i_* is the droplet diameter, *n_i_* is the number of droplets having a diameter *d_i_*, and *N* is the total number of droplets.

(3) $$span=\frac{D_{90}-D_{10}}{D_{50}}\;$$

where *D*_90_, *D*_50_, *D*_10_ are the diameters at 90%, 50%, and 10% cumulative volume.

### 2.4. Physical Stability

In order to study the kinetics of the destabilization mechanisms of these emulsions, backscattering (BS) measurements at different aging times were conducted using Turbiscan Lab Expert (Formulaction, Worthington, OH, USA). Furthermore, Turbiscan Stability Index (TSI) was used to compare the stability of the systems studied. It is a parameter defined by:

(4) $$TSI=\underset{j}{\sum }\left|scan_{ref\;}\left(h_{j}\right)-scan_{i}\left(h_{j}\right)\right|$$

where *scan_ref_* and *scan_i_* are the initial transmission value and the transmission value at a specific time, respectively, and *h_j_* is a specific height in the measuring cell.

### 2.5. Rheological Characterization

Rheological experiments were conducted using an AR 2000 rheometer (TA Instruments, Newcastle, DE, USA). The geometry used was a 60 mm serrated plate-plate geometry. Frequency sweeps were carried out from 20 to 0.05 rad/s at a stress in the linear viscoelastic range at 20 °C. The latter was analyzed by means of stress sweeps at 0.1, 1, and 3 Hz. Finally, flow curves were performed using a stress-based protocol.

### 2.6. Field Emission Scanning Electron Microscopy (FESEM)

The sample studied by FESEM was prepared following the protocol reported by Santos et al., 2020 [28]. First, the sample was fixed with glutaraldehyde (4 wt.%, cacodylate 0.1M) and osmium tetroxide (1 wt.%, cacodylate 0.1M). Subsequently, the sample was dehydrated using ethanol and acetone. Then, the sample was dried using a critical point dryer (CPD; Leica EM CPD 300, Wetzlar, Germany) for 2 h and 30 min.

### 2.7. Statistical Analysis

All laser diffraction and rheological tests were carried out using three samples, and then the mean and standard deviation of each sample were calculated. Excel software was used for analysing the data. Significant differences between sample means (*p* < 0.05) were established using a one-way ANOVA test (Tukey test).

## 3. Results and Discussion

### 3.1. Regarding the Processing Parameters

**Figure 1:**
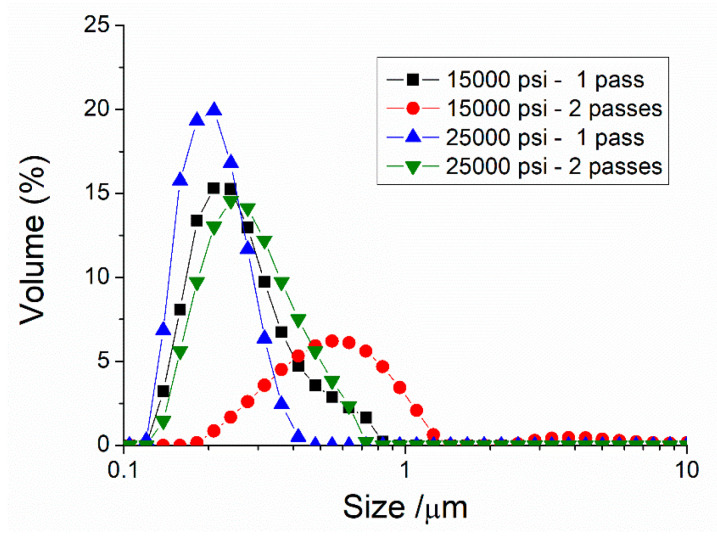
Droplet size distributions for a 50/50 system as a function of the processing parameters studied.

First of all, the influence of the two homogenization pressures and the number of passes through the microfluidizer is shown in Figure 1, for a 50/50 ratio of surfactants (50 wt.% Tween 80/50 wt.% Span 80). Interestingly, there is a reduction of droplet sizes from 15,000 to 25,000 psi. However, the droplet diameters shift towards bigger sizes from one to two passes, regardless of the homogenization pressure. These two facts suggest that the energy supplied at 25,000 psi is enough to obtain nanoemulsions (*D*_3.2_ = 192 nm), and that more than this provokes the undesirable over-processing phenomenon that leads to recoalescence. This has been previously reported in other studies using a microfluidizer with a high-pressure valve homogenizer [29,30]. Furthermore, the nanoemulsion obtained at 25,000 psi (one pass) shows a span value of 0.723 (Table 1). This value brings to light the high monodispersity of this system.

### 3.2. Regarding the Tween 80/Span 80 Ratio

**Figure 2:**
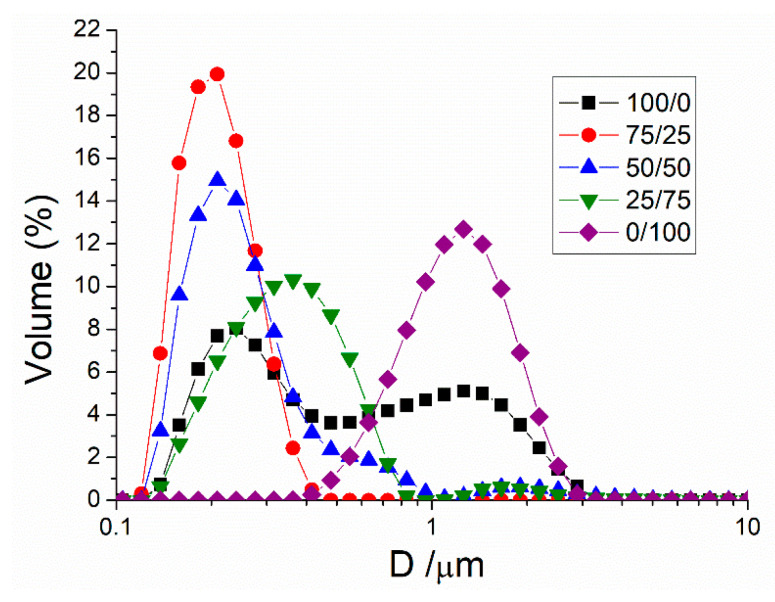
Droplet size distributions for ecological mandarin essential oil emulsions as a function of Tween80/Span80 ratio.

Figure 2 illustrates the droplet size distribution for mandarin emulsions as a function of Tween80/Span80 ratio. The Tween pure system (HLB: 15) shows a very wide distribution, with two peaks merged. Various studies have reported the instability provoked by wide droplet size distributions [31,32]. A monomodal distribution was presented by the Span pure system (HLB = 4.3). Nevertheless, this distribution is centered at about 1 micron, very far from nanosize scale. On the other hand, 50/50 (HLB = 9.7) and 25/75 (HLB = 7) display droplet size distributions with two peaks: one centered below 1 micron and the second after this value. Emulsions with bimodal DSD are more susceptible to undergoing creaming or coalescence [33]. Finally, the 75/25 (HLB = 12.3) system shows a monomodal distribution centered at about 300 nm.

Sauter diameter, volumetric diameter, and span values are illustrated in Table 2. The occurrence of Tween provokes the reduction of the Sauter diameter to nanometer scale. This supports the idea that the HLB value of 15 is not adequate for o/w emulsions [34,35]. On the other hand, emulsions with pure surfactants show values of span >1. However, the presence of the two surfactants reveals a decrease in obtaining a span value of 0.95 (<1) for the 75/25 system. Taking into account span values as well as Sauter and volumetric diameters, a 75/25 system could have the best stability since it shows the lowest polidispersity and smallest droplet diameter. Not only low droplet diameters but also low span values play an important role in maintaining the physical stability of emulsions [31].

However, according to the HLB theory reported by Griffin, a mixture of surfactant with a final HLB value between 9 and 12 is adequate to prepare stable o/w emulsions [34]. Hence, it seems that 50/50 (HLB: 9.7) and 75/25 (HLB: 12.3) systems could show the best physical stability. In addition, the optimal HLB of surfactants will be closer to the HLB value of the oil phase [36].

**Figure 3:**
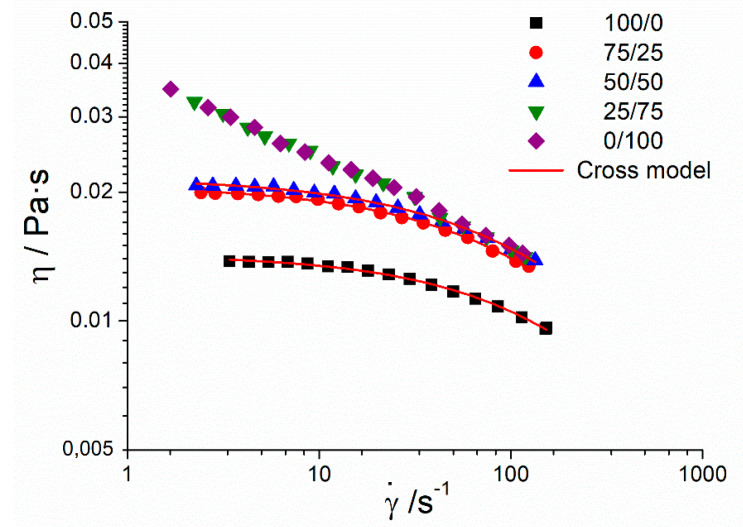
Influence of Tween80/Span80 ratio on flow behavior for ecological mandarin emulsions.

The flow behavior of emulsions formulated with different Tween80/Span80 ratios is presented in Figure 3. All emulsions exhibited a decrease of viscosity (*ƞ*), with a shear rate ($\dot{\gamma }$) characteristic of shear-thinning behavior. However, the highly-concentrated system of Tween and the 50/50 systems showed a viscosity with a trend to reach a plateau value at low shear rates, and lower viscosities, than the 25/75 and 0/100 systems. This tendency could fit with the Cross model while the 25/75 and 0/100 systems did not present this behavior. Cross fitting parameters are shown in Table 3: zero-shear viscosity (*ƞ*_0_), the inverse of critical shear rate (*k*), and m that is related to the flow index. In addition, the viscosity increases when Sauter diameter decreases in the highly-concentrated Tween and 50/50 systems. The same tendency can be observed between the 0/100 and 25/75 systems.

**Figure 4:**
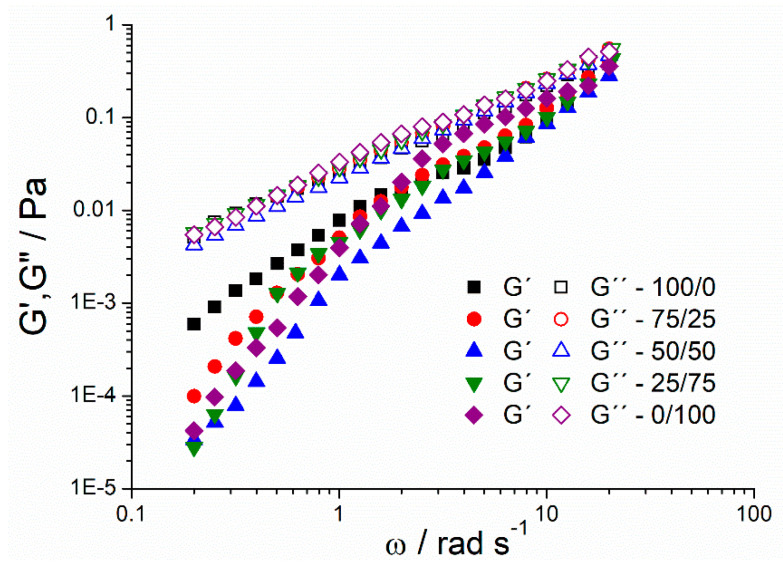
Mechanical spectra for ecological mandarin emulsions as a function of Tween/Span ratios.

Figure 4 shows the mechanical spectra of the mandarin emulsions studied. Interestingly, all emulsions presented G″ (viscous modulus) values higher than G′ (storage modulus) values in all the frequency range (ω). In addition, the slope of G′ is high. These facts are related to viscoelastic liquids; these systems are not gels. The same behavior illustrated could be due to the presence of 0.25 wt.% of guar gum in all the systems. The elastic and viscous responses of the emulsions seemed to be governed by the presence of the guar gum. This fact has previously taken place in emulsions containing guar gum and xanthan gum [37,38].

**Figure 5:**
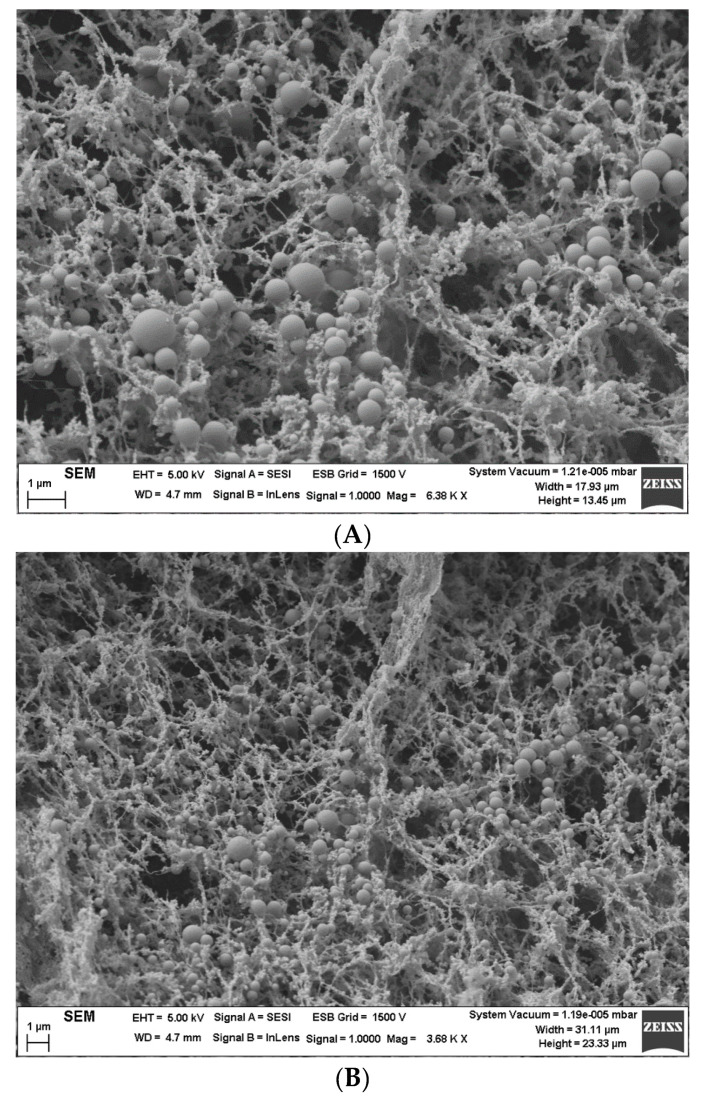
(**A**) Microstructure observed by FESEM for the 75/25 system, (**B**) Microstructure observed by FESEM for the 50/50 system.

Figure 5A shows the microstructure formed by mandarin oil droplets and guar gum in a 75/25 system, by way of example. Interestingly, aggregations of droplets can be observed. These flocs seem to be inserted in a complex network formed by guar gum. This is the reason why the gum governs the dynamical rheological properties of these emulsions. The droplet size does not significantly influence the oscillatory response. However, when a flow is applied, the microstructure can be broken, showing different flow behaviors. All the systems studied show very similar microstructures. Figure 5B illustrates the microstructure of the 50/50 system. Microstructural differences between 50/50 and 75/25 emulsion cannot be observed.

**Figure 6:**
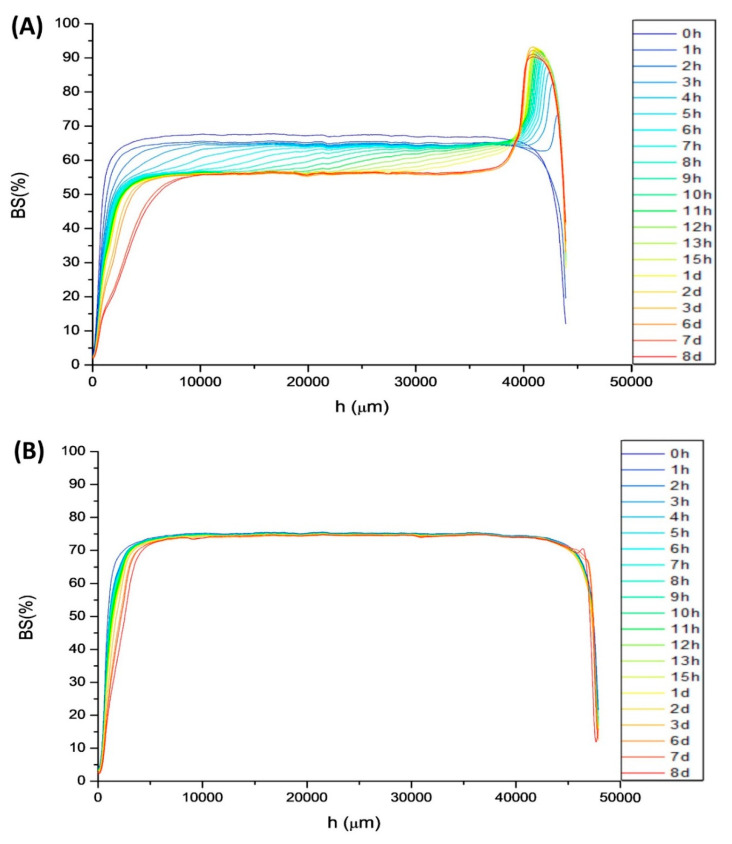
Backscattering values versus measuring cell height with aging time for (**A**) the 100/0 system and (**B**) the 75/25 system.

The Backscattering (BS) versus height of the measuring cell (h) of the 100/0 systems at aging time is illustrated in Figure 6A. The profiles of 50/50, 25/75, and 0/100 are quite similar. There is a decrease of BS in the low and in the middle zone of the measuring cell. The former is related to a creaming process; droplets go to the upper zone of the measuring cell clarifying the low zone. The latter is provoked by a growth of droplet sizes. These two destabilization mechanisms provoke the increase of BS in the upper zone of the measuring cell; the mandarin oil appears in a fine layer (oiling off). These destabilization processes could be provoked by the bigger droplet sizes and/or higher polidispersity [31]. Although the 25/75 and 50/50 systems underwent the same mechanisms, these were to a lower degree. This could be explained by the differences between droplet size distributions.

On the other hand, the 75/25 system did not show any variance of BS in the middle and the upper zone of the measuring cell (Figure 6B). Hence, coalescence or Ostwald ripening is not taking place, conversely to the other systems studied. However, creaming was undergone in the first 8 days of aging time. This mechanism could be inhibited increasing the gum concentration.

**Figure 7:**
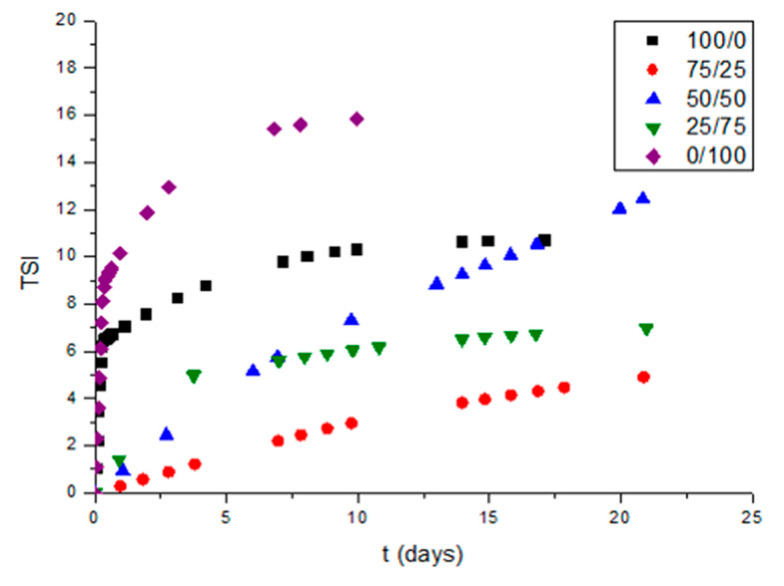
Values of Turbiscan Stability Index (TSI) with aging time for mandarin nanoemulsions as a function of Tween80/Span80 ratio.

In order to get a deeper insight into the physical stability of these systems, Figure 7 is shown. This figure illustrates the Turbiscan Stability Index (TSI), with aging time as a function of surfactant ratio. TSI is a measure of all the destabilization mechanisms. Higher values of TSI are related to poorer physical stabilities. This representation proves the best stability was obtained by the 75/25 system. This fact could be related to the lower droplet sizes and the lower span value of this emulsion. These values may inhibit the growth of droplet sizes that the other systems presented. Hence, the more adequate HLB value for preparing stable ecological mandarin emulsions is 12.3.

## 4. Conclusions

Mandarin essential oils were successfully incorporated as dispersed phase in stable food-grade emulsions formulated with guar gum. In this study, the mean droplet diameters and physical stability of mandarin oil-in-water emulsions were found to be improved by optimizing the emulsification method and HLB of a mixture of food-grade surfactants. First, the influence of homogenization pressure, and the number of cycles, on droplet size distribution was characterized by laser diffraction measurements. These measurements showed the best results for those emulsions developed by microfluidization at the highest homogenization pressure (25,000 psi) and one cycle. Then, the influence of different Tween80/Span80 ratios was investigated. HLB exerted a marked influence on the droplet size distributions, rheological properties, and physical stability of emulsions. All emulsions exhibited shear thinning flow properties. The mechanical spectra corresponding to these emulsions were typical of liquid dispersions since G″ is dominant over G′. The dynamic rheological properties may be governed by the presence of guar gum. The most stable emulsion was the one prepared with an HLB value of 12.3 (75 wt.% Tween80/25 wt.% Span80). This emulsion presented high physical stability due to its nanometric droplet size and low polidispersity. Overall, our results show that these systems can have multiple potential applications as natural food additives and delivery systems.

## Figures and Tables

**Table 1:** Sauter diameter (*D*_3,2_), volumetric diameter (*D*_4,3_), and span values for a 50/50 system as a function of processing parameters. a–c Mean value ± standard deviation; values without common superscripts within the same column were significantly different (*p* < 0.05).

Processing Parameters Used	D_3,2_ (µm)	D_4,3_ (µm)	Span
**15,000 psi—1 pass**	0.227 ± 0.018 ^a,c^	0.263 ± 0.013 ^a,d^	1.179 ± 0.071 ^a,c^
**15,000 psi—2 passes**	0.866 ± 0.065 ^b^	0.441 ± 0.022 ^b^	1.320 ± 0.080 ^a^
**25,000 psi—1 pass**	0.192 ± 0.011 ^a^	0.208 ± 0.011 ^c^	0.723 ± 0.041 ^b^
**25,000 psi—2 passes**	0.246 ± 0.019 ^c^	0.279 ± 0.012 ^d^	1.066 ± 0.062 ^c^

**Table 2:** Influence of Tween80/Span80 ratio on Sauter diameter (*D*_3,2_), volumetric diameter (*D*_4,3_), and span values. a–e Mean value ± standard deviation; values without common superscripts within the same column were significantly different (*p* < 0.05).

Tween80/Span80 Ratio	D_3,2_ (µm)	D_4,3_ (µm)	Span
**100/0**	0.367 ± 0.025 ^a^	0.691 ± 0.042 ^a^	3.063 ± 0.153 ^a^
**75/25**	0.192 ± 0.012 ^b^	0.208 ± 0.012 ^b^	0.723 ± 0.036 ^b^
**50/50**	0.232 ± 0.020 ^b^	0.275 ± 0.015 ^c^	1.342 ± 0.067 ^c^
**25/75**	0.308 ± 0.020 ^c^	0.553 ± 0.032 ^d^	1.752 ± 0.088 ^d^
**0/100**	1.043 ± 0.081 ^d^	1.315 ± 0.079 ^e^	1.590 ± 0.080 ^e^

**Table 3:** Cross model fitting parameters of ecological mandarin emulsions as a function of Tween80/Span80 ratio. Zero-shear viscosity (*ƞ*_0_), the inverse of critical shear rate (*k*), and m that is related to the flow index. a–c mean value ± standard deviation; values without common superscripts within the same column were significantly different (*p* < 0.05).

Tween80/Span80 Ratio	*η*_0_ (Pa·s)	*k* (s)	m
**100/0**	0.0143 ± 0.0011 ^a^	0.010 ± 0.001 ^a^	0.78 ± 0.02 ^a^
**75/25**	0.0207 ± 0.0015 ^b^	0.015 ± 0.001 ^b^	0.74 ± 0.01 ^b^
**50/50**	0.0216 ± 0.0014 ^b^	0.016 ± 0.001 ^b^	0.72 ± 0.01 ^b^
